# Supplementary material for: Marine Cyanobacteria as Sources of Lead Anticancer Compounds: A Review of Families of Metabolites with Cytotoxic, Antiproliferative, and Antineoplastic Effects
Source: Molecules. 2022 Jul 27;27(15):4814. doi: 10.3390/molecules27154814 (PMC9369884; doi:10.3390/molecules27154814)
Supplement: Supplementary file 1 [file molecules-27-04814-s001.zip › Supp. Figures S1-S3.pdf]

molecules

MDPI

Supplementary Materials

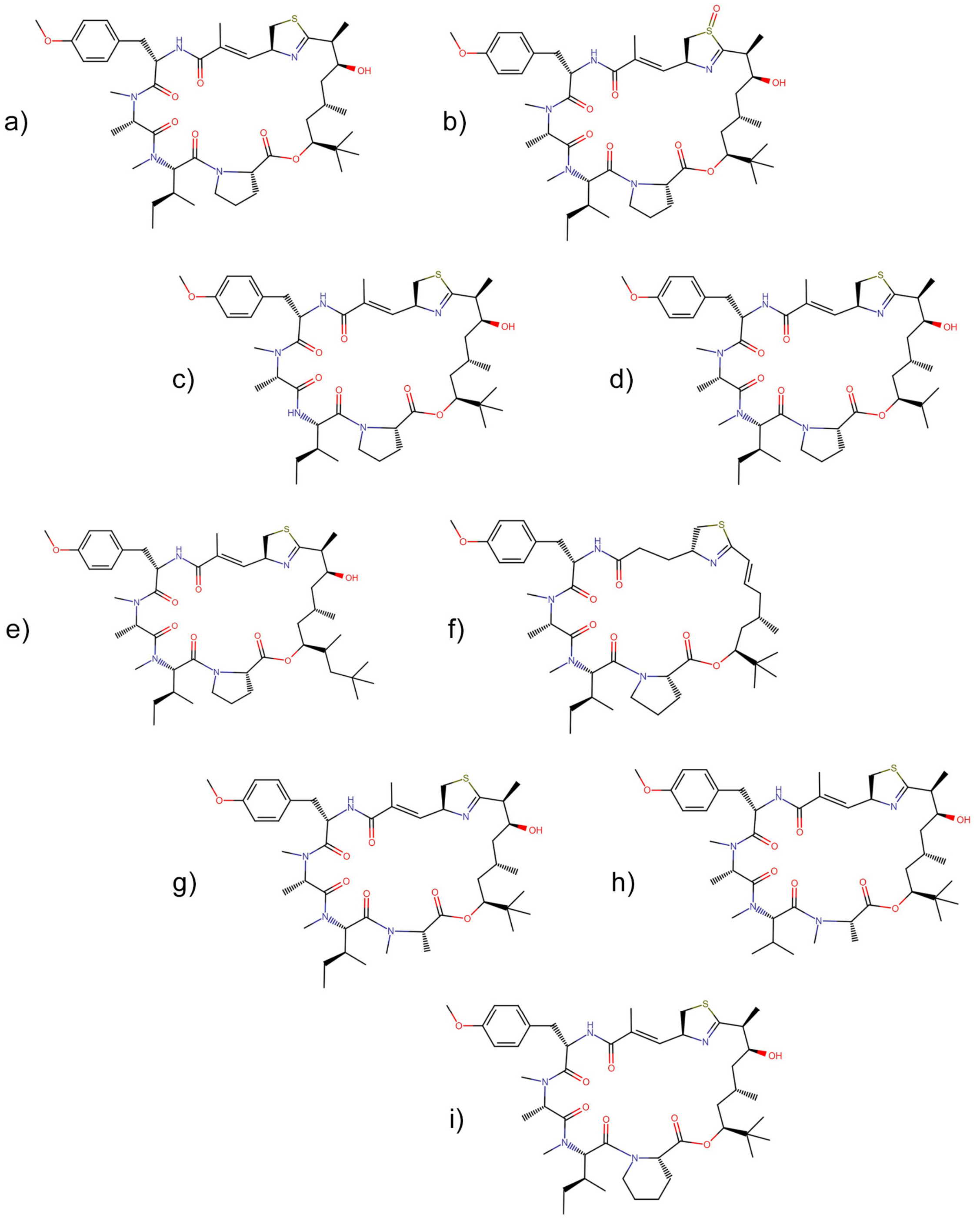

Figure S1. Apratoxin family. a) apratoxin A; b) apratoxin A sulfoxide; c) apratoxin B; d) apratoxin C; e) apratoxin D; f) apratoxin E; g) apratoxin F; h) apratoxin G; i) apratoxin H.

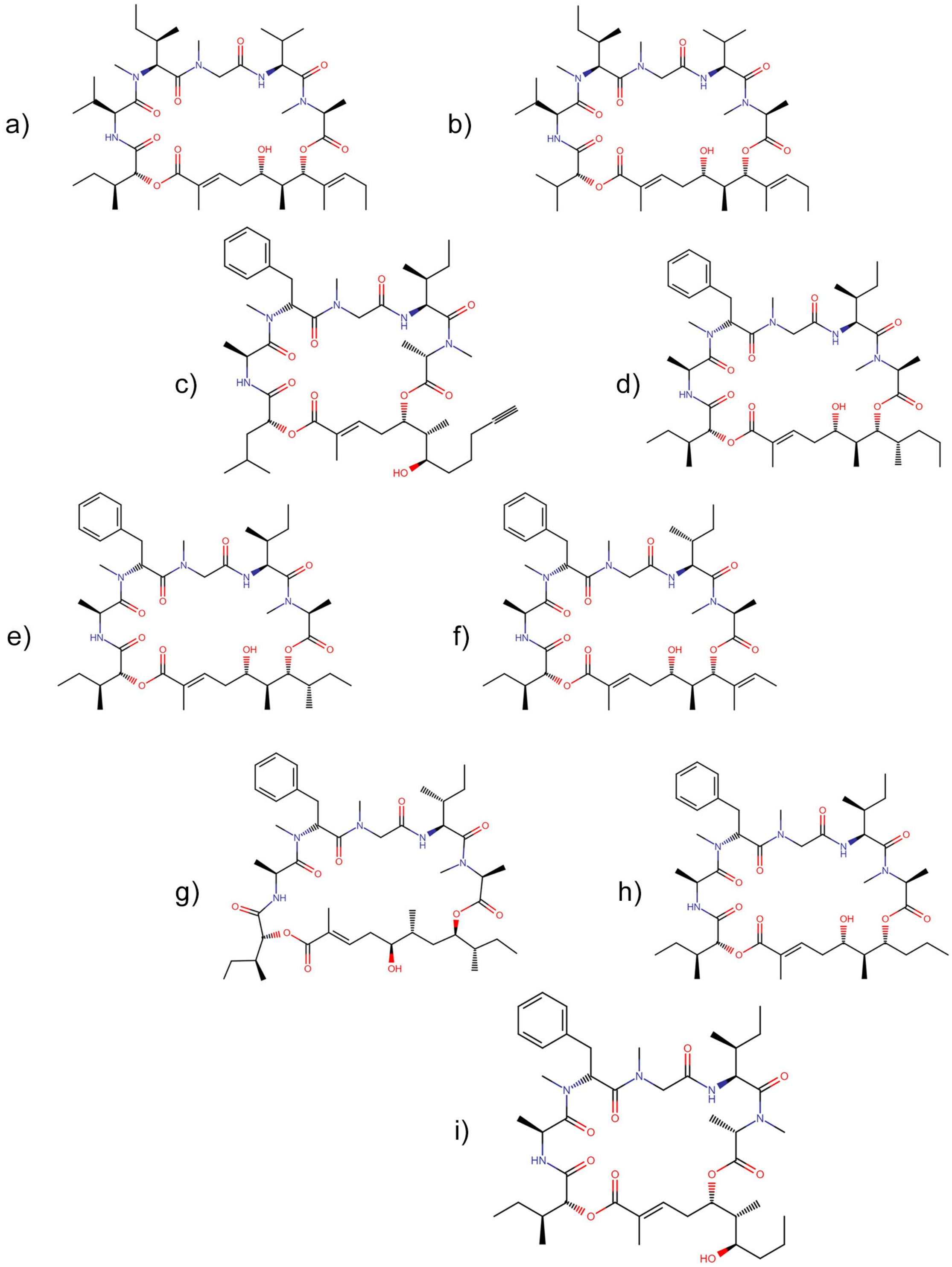

Figure S2. Aurilide family. a) aurilide B; b) aurilide C; c) Palau'amide; d) odoamide; e) lagunamide A; f) lagunamide B; g) lagunamide C; h) lagunamide D; i) lagunamide D'.

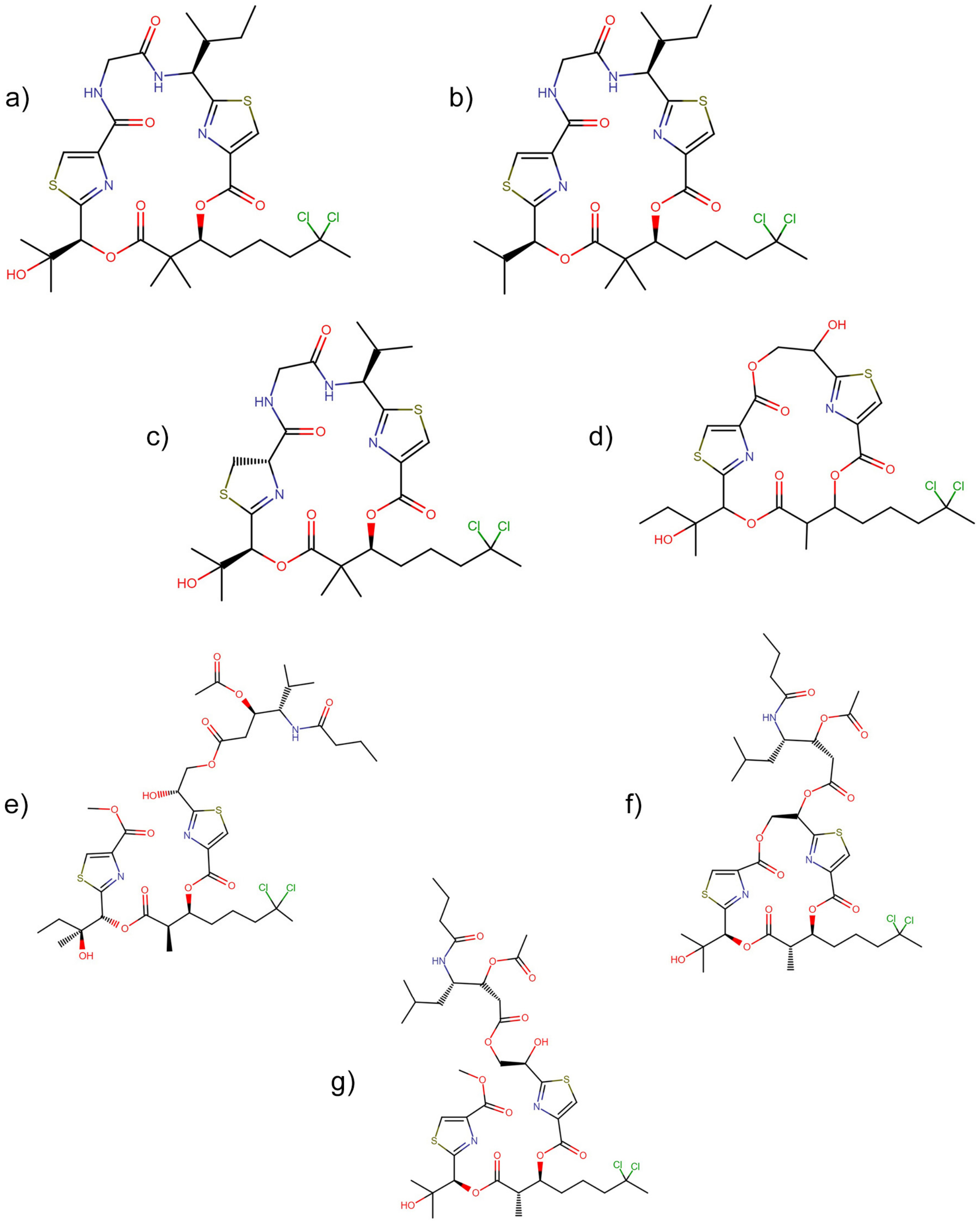

Figure S3. Lyngbyabellin family. a) lyngbyabellin A; b) 27-deoxylyngbyabellin A; c) lyngbyabellin B; d) lyngbyabellin C; e) lyngbyabellin D; f) lyngbyabellin E; g) lyngbyabellin F.

h)

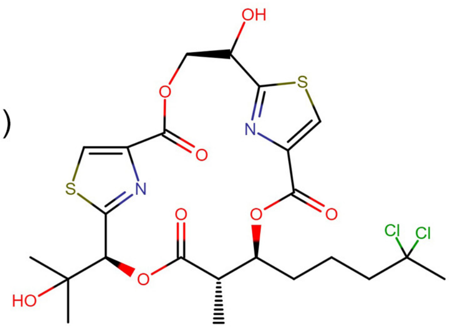

i)

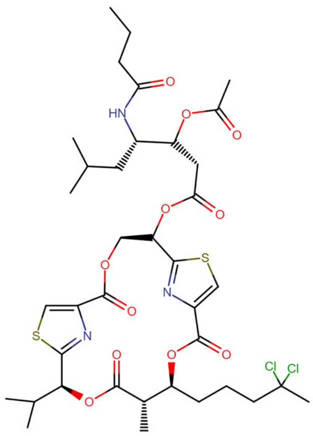

j)

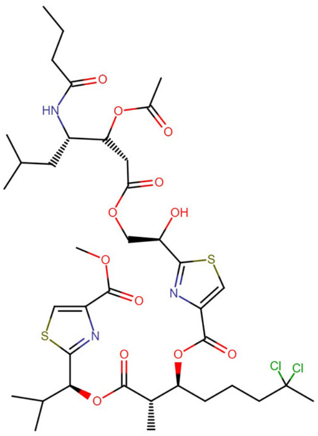

k)

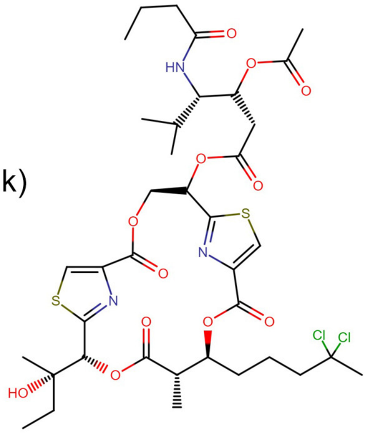

l)

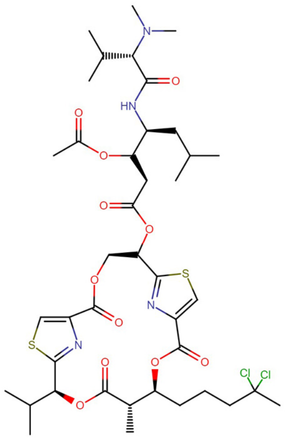

m)

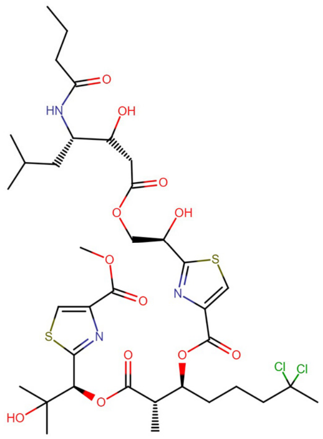

Figure S3 (cont). h) lyngbyabellin G; i) lyngbyabellin H; j) lyngbyabellin I; k) lyngbyabellin J; l) lyngbyabellin N; m) lyngbyabellin P.
