# Supplementary material for: Marine Cyanobacteria as Sources of Lead Anticancer Compounds: A Review of Families of Metabolites with Cytotoxic, Antiproliferative, and Antineoplastic Effects
Source: Molecules. 2022 Jul 27;27(15):4814. doi: 10.3390/molecules27154814 (PMC9369884; doi:10.3390/molecules27154814)
Supplement: Supplementary file 1 [file molecules-27-04814-s001.zip › Supp. Table S2.pdf]

molecules

Supp. Table S2. Tumor cell lines origin

Cell line	Cancer type
A549	Human lung carcinoma
NCI-H460	
NCI-H82	
NCI-H446	
NCI-H69	
NCI-H510	
NCI-H187	
NCI-H125	
L78	
SPC-A1	
95D	
PC-10	
PC-13	
QG-56	
NCI-H1975	Human lung adenocarcinoma
GLC-82	
PC-3	
PC-8	
PC-9	
NCI-H466	Human lung cancer
SNB-75	Human glioblastoma
U87-MG	
SF-295	
SF-268	
U251N	
IMR-32	Human neuroblastoma
SH-SY5Y	
Neuro-2a	Mouse neuroblastoma
RKO	Human colon carcinoma
HCT-116	
HT-29	Human colon adenocarcinoma
HCT8	
KM20L2	
MAWI	
SW480	
SW620	
LoVo	
BE	Human colon cancer
SK-MEL-2	Human melanoma
SK-MEL-5	
LOX IMVI	
1976	
3163	
3629	
3670	
3682	
3702	
M375	

Cell line	Cancer type
MDA-MB-435	Human melanoma (previously described as human breast carcinoma)
HeLa<br>HeLa S_3	Human cervical carcinoma
SK-OV-3<br>OVCAR-3	Human ovarian adenocarcinoma
A2780<br>CHI<br>41M<br>HX/62	Human ovarian cancer
MDA-MB-231<br>MDA-MB-436<br>MDA-MB-468<br>MCF7	Human breast adenocarcinoma
HS578T<br>BT474<br>T-47D	Human breast carcinoma
NCI/ADR (previously known as the MCF7/ADR)	Human drug-resistant breast cancer
PC3<br>BxPC-3<br>LNCap	Human prostate adenocarcinoma
DU-145	Human prostate carcinoma
HEC-6	Human endometrial adenocarcinoma
KB	Human papilloma (previously described as human nasopharyngeal carcinoma)
Bel 7402	Human papilloma (previously described as human liver carcinoma)
BJ	Human foreskin fibroblast
BJ Shp 53	Human foreskin fibroblast (p53 knocked down)
CA46<br>Daudi	Human Burkitts lymphoma
U937<br>DB<br>SCLHT<br>RL<br>SR	Human lymphoma
P388	Mouse lymphoma
CEM<br>HL-60	Human leukemia
L1210	Mouse leukemia
RPMI-8226	Human plasmacytoma
797<br>10326	NUT midline carcinoma
769-P<br>786-O	Human renal adenocarcinoma
Eca-109	Human esophageal carcinoma
MKN74	Human gastric tubular adenocarcinoma
KATO-III	Human gastric adenocarcinoma
MG-63	Human osteosarcoma

Cell line	Cancer type
U2OS	
HT-1080	Human fibrosarcoma
PANC-1<br>MiaPaCa	Human pancreatic carcinoma
T-24	Human bladder carcinoma
HepG2<br>HuH-7	Human liver carcinoma
JFCR39 panel	Panel of 39 human cancer cell<br>Lines at the Japanese Foundation for<br>Cancer<br>Research
NCI 60	NCI-60 Human tumor cell lines screen
